# Resibufogenin and Cinobufagin Activate Central Neurons through an Ouabain-Like Action

**DOI:** 10.1371/journal.pone.0113272

**Published:** 2014-11-24

**Authors:** Ze-Jun Wang, Liqin Sun, Thomas Heinbockel

**Affiliations:** Department of Anatomy, College of Medicine, Howard University, Washington, DC, United States of America; Universidade Federal do Rio de Janeiro, Brazil

## Abstract

Cinobufagin and resibufogenin are two major effective bufadienolides of Chan su (toad venom), which is a Chinese medicine obtained from the skin venom gland of toads and is used as a cardiotonic and central nervous system (CNS) respiratory agent, an analgesic and anesthetic, and as a remedy for ulcers. Many clinical cases showed that Chan su has severe side-effects on the CNS, causing shortness of breath, breathlessness, seizure, coma and cardiac arrhythmia. We used whole-cell recordings from brain slices to determine the effects of bufadienolides on excitability of a principal neuron in main olfactory bulb (MOB), mitral cells (MCs), and the cellular mechanism underlying the excitation. At higher concentrations, cinobufagin and resibufogenin induced irreversible over-excitation of MCs indicating a toxic effect. At lower concentrations, they concentration-dependently increased spontaneous firing rate, depolarized the membrane potential of MCs, and elicited inward currents. The excitatory effects were due to a direct action on MCs rather than an indirect phasic action. Bufadienolides and ouabain had similar effects on firing of MCs which suggested that bufadienolides activated neuron through a ouabain-like effect, most likely by inhibiting Na^+^/K^+^-ATPase. The direct action of bufadienolide on brain Na^+^ channels was tested by recordings from stably Na_v_1.2-transfected cells. Bufadienolides failed to make significant changes of the main properties of Na_v_1.2 channels in current amplitude, current-voltage (I-V) relationships, activation and inactivation. Our results suggest that inhibition of Na^+^/K^+^-ATPase may be involved in both the pharmacological and toxic effects of bufadienolide-evoked CNS excitation.

## Introduction

Chan su is a traditional Chinese medicine prepared from the dried white secretion of the auricular glands and the skin glands of *Bufo bufo gargarizans Cantor* or *Bufo melanostictus Schneider*. It is traditionally used as a cardiotonic agent, CNS respiratory stimulant, analgesic, anesthetic and as remedies for ulcer boils, scrofula, sore throat, and cancer for hundreds of years in China and in most eastern countries in Asia. It is also the major component of such popular traditional Chinese medications as Lu-Shen-Wan [1], She-Xiang-Bao-Xin-Wan [2], and Kyushin [1]. These medicines are used as remedies for tonsillitis, sore throat, furuncle, palpitations, and as a cardiotonic agent for stimulation of myocardial contraction [3]–[5] and as analgesic for pain relief [6].

The major active components in chan su are bufadienolides such as bufalin, cinobufagin and resibufogenin. Bufadienolides are a group of steroid hormones. They inhibit the adenosine triphosphatase sodium-potassium pump, Na^+^/K^+^-ATPase [7]–[14]. Like other cardiac glycosides such as digoxin, bufadienolides cause an increase in sodium excretion, produce vasoconstriction resulting in hypertension, and act as cardiac inotropes [7]. The chemical structure of several toad-derived bufadienolides has similarities to digoxin/digitalis with similar cardiotoxic and lethal effects. The adverse drug effects of digoxin, which is used to treat various heart conditions, are concentration-dependent and the result of competition of digoxin with K^+^ ions for the same binding site on the Na^+^/K^+^-ATPase pump.

Recent studies showed that bufadienolides display many pharmacological actions such as anti-inflammation [15], [16], enhancement of cardiac contractility [17], inhibition of tumors by inducing apoptosis [18], [19]. It is thought that the Na^+^/K^+^-ATPase participates in these cardiotonic and anti-tumor actions.

Chan su and Chan su-containing medicines such as Liu-Shen-Wan display pharmacological and toxic effects on the peripheral and central nervous system in clinical cases and *in vivo* experiments [20], [21], [6]. Chansu and bufadienolides show properties of anesthetics, analgesics, and activate the CNS respiratory system [20], [22]–[24]. Topical administration of Chan su results in strong anesthetic effects [22]. Many clinical cases have reported that high dosages of either Liu-Shen-Wan or Chan su cause severe side-effects such as cardiac arrhythmia, over-excitation of the respiratory system and breathlessness, seizure, and coma [25]–[27], [19]. A fatal case has been reported of a Chinese woman after ingestion of Chinese herbal tea that contained Chan su [28]. Evidence from the clinic and behavioral experiments *in vivo* have shown that Liu-Shen-Wan or Chan su are toxic for the heart and CNS [22]. An overdose of bufadienolides or Liu-Shen-Wan results not only in cardiac arrhythmia by modifying the functions of both cardiomyocytes and Purkinje fibers of the heart [21], [27], [29], but also evokes epileptic seizures [30], acute shortness of breath, muscle cramps, and finally death by paralysis by affecting the CNS [22], [29], [31]. While the effects of chan su and bufadienolides on the cardiac system have been widely studied, the pharmacological and toxic effects of bufadienolides on neurons in the CNS are unknown. Here, we hypothesize that Chan su and bufadienolides activate neurons in the CNS, and that neuronal activation by and excitotoxic effects of bufadienolides are mediated by the Na^+^/K^+^-ATPase pump. The involvement of Na^+^/K^+^-ATPase in activation of neurons could, therefore, be the basis for the neuro-pharmacological and toxic effects of Chan su.

**Figure 1:**
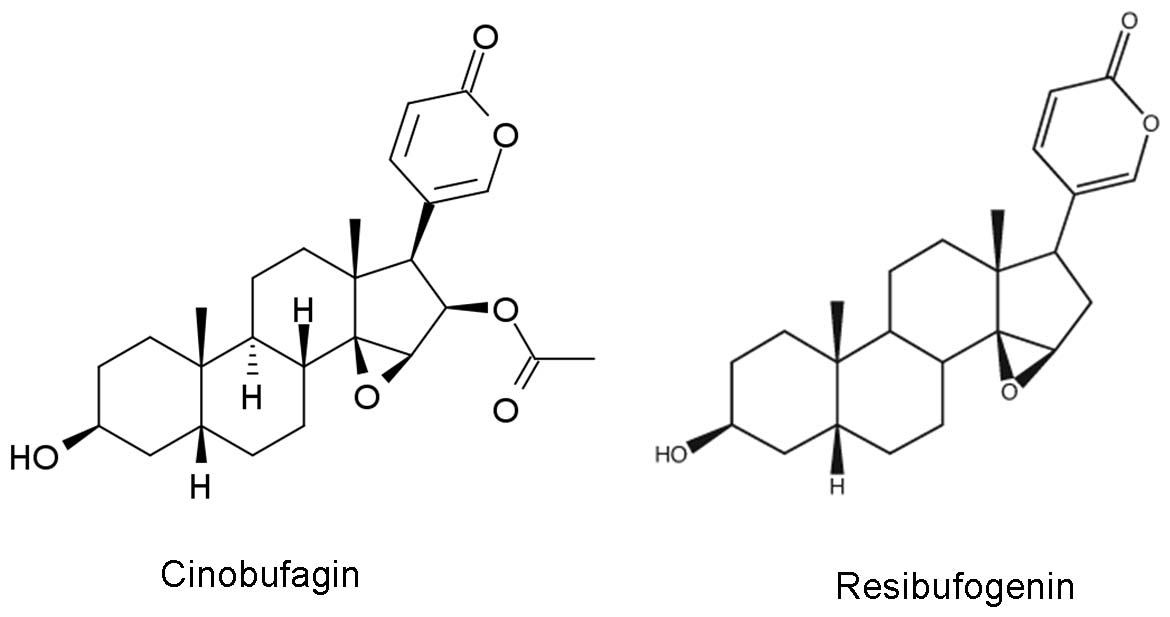
Chemical structures of cinobufagin and resibufogenin.

Cinobufagin and resibufogenin are the major active bufadienolides isolated from Chan su (chemical structures are illustrated in Fig. 1). Resibufogenin was recently shown to activate voltage-gated Na^+^ channels but to reduce the firing rate of evoked repetitive firing in cultured rat hippocampal neurons [32]. A reduction of evoked firing rate appears to contrast sharply with the reported toxic effects of Chan su since symptoms of Chan su-mediated toxic effects in the clinic suggest an excitatory action on neurons. MCs are principal neurons and play a crucial role in processing sensory information in the mouse MOB. They receive direct synaptic inputs from the axons of olfactory receptor neurons, send excitatory projections to olfactory cortical areas, and receive strong feedback inhibition primarily through reciprocal dendrodendritic synapses with local interneurons [33], [34]. MCs in the MOB display their neuronal activity as spontaneous action potential firing with a frequency of 1–6 Hz. The excitability of MCs can be modulated by intrinsic membrane receptors as well as synaptic inputs [33]–[36]. Thus, MCs are an advantageous cell type to explore their possible interaction with bufadienolides. Here, we used electrophysiological recordings from MCs in acute slice preparations of the mouse MOB and from transfected cells expressing Na^+^ channels to determine the effects of bufadienolides on neuron activity, and the possible mechanisms underlying the pharmacological and toxic effects of bufadienolides or Chan su.

## Experimental Procedures

### Animals and Slice Preparation

The experiments were approved by the Howard University Animal Care and Use Committee. Wild type mice (C57BL/6J, Jackson Laboratory, Bar Harbor, ME; 16–25 day old) were euthanized and, subsequently, we harvested tissue for in vitro brain slices. Upon achieving a deep surgical plane of anesthesia (isofluorane), the head was removed by decapitation. The brain was rapidly dissected and immersed in artificial cerebrospinal fluid (ACSF, see below) at 4°C, as previously described [36]. Horizontal slices (400 µm-thick) were cut parallel to the long axis using a vibratome (Vibratome Series 1000, Ted Pella Inc., Redding, CA). After 30 min at 30°C, slices were incubated in a holding bath at room temperature (22°C) until use. For recording, a brain slice was placed in a recording chamber mounted on a microscope stage and maintained at 30±0.5°C by superfusion with oxygenated ACSF flowing at 2.0–2.5 ml/min.

### Slice Recording and Data Acquisition

Visually-guided recordings were obtained from cells in the mitral cell layer with near-infrared differential interference contrast optics and a BX51WI microscope (Olympus Optical, Tokyo, Japan) equipped with a camera (C2400-07, Hamamatsu Photonics, Japan). Images were displayed on a Sony Trinitron Color Video monitor (PVM-1353MD, Sony Corp. Japan). Recording pipettes (5–8 MΩ) were pulled on a Flaming-Brown P-97 puller (Sutter Instrument Co., Novato, CA) from 1.5 mm O.D. borosilicate glass with filament. Seal resistance was routinely>1 GΩ and liquid junction potential was 9–10 mV with negative charges inside of electrode; reported measurements were not corrected for this potential. Data were obtained using a Multiclamp 700B amplifier (Molecular Devices, Sunnyvale, CA). Signals were low-pass Bessel filtered at 2 kHz and digitized on computer disc (Clampex 10.1, Molecular Devices). Data were also collected through a Digidata 1440A Interface (Molecular Devices) and digitized at 10 kHz. Holding currents were generated under control of the Multiclamp 700B Commander.

The ACSF consisted of the following (in mM): 124 NaCl, 3 KCl, 2 CaCl_2_, 1.3 MgSO_4_, 10 glucose, 4.4 sucrose, 26 NaHCO_3_, 1.25 NaH_2_PO_4_ (pH 7.4, 300 mOsm), saturated with 95 O_2_/5% CO_2_. For intracellular recording of spiking activity, the pipette-filling solution consisted of the following (in mM): 144 K-gluconate, 2 MgCl_2_, 10 HEPES, 5Mg_2_ATP, 0.5 Na_3_GTP, 2 NaCl, 0.2 EGTA. For current recording, electrodes were filled with a low-Cl¯ pipette solution containing (in mM): 125 cesium methanesulfonate (CsMeSO3), 1 NaCl, 10 phosphocreatine di-tris salt, 5 ATP, 0.5 GTP, 0.5 EGTA, 10 HEPES, 10 QX-314 [2(triethylamino)*-N-*(2,6-dimethylphenyl) bromide], pH 7.3 with 1N CsOH (290 mOsm).

### Cell Culture

The CNaIIA cell line (gift of Dr. W.A. Catterall) was derived from a CHO-K1 cell line stably transfected with a cDNA encoding the rat brain type IIA Na^+^ channel (Na_v_1.2A) [37], [38]. CNaIIA cells were cultured in RPMI medium (Gibco) with 5% fetal bovine serum, and 100 µg/ml streptomycin and 100 U/ml Penicillin. G418 (400 µg/ml) was included to select for transfectants. Then the cells were passed and plated on glass coverslips in 35-mm dishes in a 5% CO_2_ atmosphere at 37°C for 1–3 days before experimentation.

### Whole-cell Voltage-clamp Recording from Transfected Chinese Hamster Ovary (CHO) Cells

Na^+^ currents were recorded using the whole-cell patch clamp recording technique [39]. The cultured cells on coverslips were transferred to a handmade recording chamber and continuously perfused at room temperature with extracellular solution containing (in mM): 130 NaCl, 4 KCl, 1.5 CaCl_2_, 1.5 MgCl_2_, 5 Glucose, 5 HEPES, 20 Sucrose, pH 7.4 adjusted with NaOH. The recording chamber volume was approximately 0.4 ml and the flow rate was 0.6 ml/min. MP-285 micromanipulator (Sutter Instrument Co., Novato, CA) was used to place the electrode onto the cell. Patch pipettes were pulled from borosilicate glass capillaries (Drummond Scientific Co., Broomall, PA) on an electrode puller (Model P-97, Sutter Instrument Co.) and were filled with a 0.2 µm filtered internal solution containing (in mM): 90 CsF, 60 CsCl, 10 NaCl, 5 HEPES, pH 7.4 adjusted with NaOH. The pipettes had input resistance of 0.8–1.4 MΩ. Recordings were performed at room temperature (22°C) with a patch clamp EPC 9 (HEKA Elektronik GmbH, Germany) and were filtered at 5 kHz. Leakage currents were subtracted using a P/4 or P/2 protocol. Pulse (HEKA Elektronik GmbH, Germany) was used for experimental control and basic data analysis.

The methods of Na^+^ currents recorded from Na_v_1.2 expressed cells were similar to a previous report [40]. The cells with series resistance of less than 2.5 MΩ were used for further drug test experiments. Only cells with whole cell maximal Na^+^ currents of at least 1 nA were used in the analysis. Na^+^ currents recorded from Na_v_1.2A expressing cells always increased progressively within the first 20 min after establishing the whole-cell-recording configuration and then were relatively stable. Thus, drugs were applied only in this period (after 20 min), especially for testing tonic inhibition of the drug. Time-dependent shifts (∼0.5 mV before and after perfusion) in the inactivation curve of Na_v_1.2 have not been considered in calculating the shift by the drug.

### Chemicals and Drug application

Resibufogenin and cinobufagin were supplied by the National Institute for the Control of Pharmaceutical and Biological Products (Beijing, China). Stock solutions of 500 mM resibufogenin and cinobufagin was prepared in dimethyl sulfoxide (DMSO) and then diluted to the desired concentrations for experimentation. For all the experiments, the drugs were applied by perfusion (final concentration of DMSO in bath <0.1%). The following drugs were also bath applied: L-2-amino-5-phosphonopentanoic acid (AP5, APV), 6-cyano-7-nitroquinoxaline-2-3-dione (CNQX), 2-(3-carboxypropyl)-3-amino-6-(4 methoxyphenyl)-pyridazinium bromide (gabazine, SR-95531). Chemicals were supplied by Tocris (Ellisville, MO). Control recordings showed that 0.002% and 0.1% DMSO had no detectable effects on MC activity and the Na^+^ currents in transfected CHO cells, respectively.

#### Data Analysis

The data obtained with slice recording were analyzed using a combination of Clampfit 10.2 (Molecular Devices) and Origin8 (OriginLab Corporation, Northampton, MA). The data obtained with transfected cells were analyzed using a combination of PulseFit (HEKA Elektronik GmbH, Germany) and SigmaPlot 9.0 (Jandel Scientific, Corte Madera, CA) software. All results are presented as the mean ± S.E.M. Tests for statistical significance were performed using paired Student's *t*-tests, and one-way ANOVA followed by the Bonferroni test for multiple comparisons.

## Results

Recordings were obtained from 76 MCs with whole-cell recordings in mouse MOB slices. All the recorded MCs showed measurable responses to cinobufagin and resibufogenin. MCs were identified visually by their soma location and relatively large soma size, and by their input resistance (297±19.2 MΩ, *n* = 46). In transfected cells, 20 to 30% of Na_v_1.2-expressing CHO cells displayed fast, transient inward currents following depolarization. The maximal peak inward currents of Na_v_1.2 ranged from 0.7 to 8 nA. However, in a few cells, it went up to 20 nA. These currents could be blocked completely by 0.5 µM tetrodotoxin (TTX), confirming their identity as uncontaminated Na^+^ currents under these recording conditions.

### Higher concentration of both cinobufagin and resibufogenin irreversibly activated mitral cells

MCs are principal neurons and play a crucial role in processing sensory information in MOB. In slices, MCs generate intrinsic spontaneous action potentials (1–6 Hz) [35], [36]. Here, we took advantage of the intrinsic properties of MCs such as spontaneous firing, membrane potential and membrane conductance to test the effect of bufadienolides on neuronal activity in CNS neurons and to determine the underlying cellular mechanism of the action of bufadienolides.

**Figure 2:**
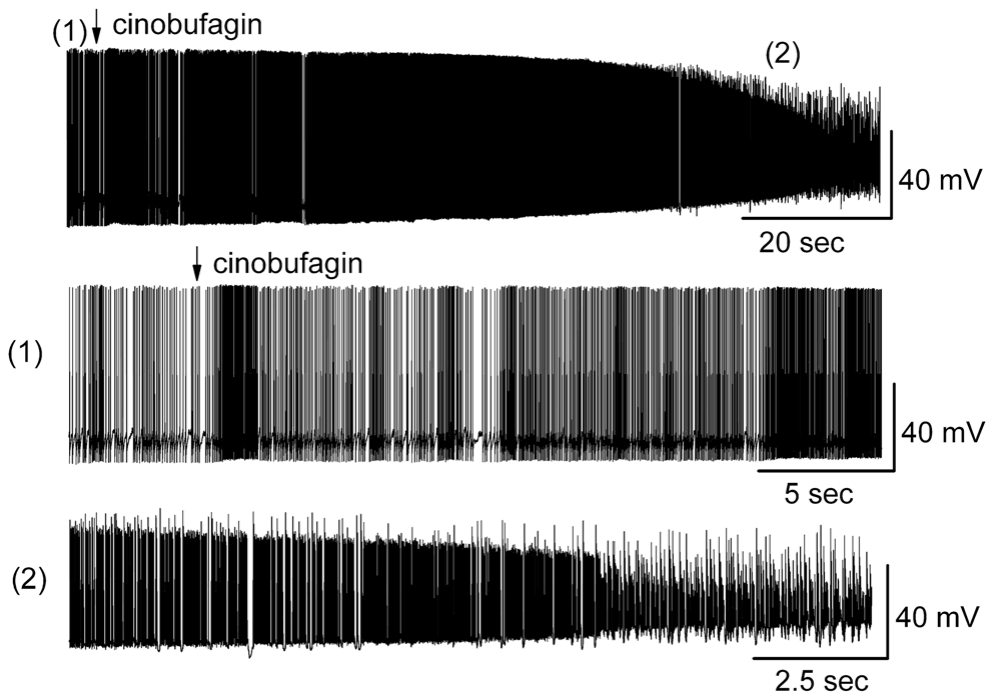
Over-excitation of MC evoked by cinobufagin. Arrow indicates the time point of adding 10 µM cinobufagin. The recording trace at the (1) and (2) time point in the upper trace are shown at an extended time scale in the middle trace and lower trace.

Bath application of 10 µM cinobufagin activated MCs irreversibly, evoking a lasting increase in depolarization and firing rate of MCs (Fig. 2). MCs that were over-excited by 10 µM cinobufagin exhibited difficulty to recover from the strong depolarization and some of the recorded MCs subsequently died. Cinobufagin strongly depolarized MCs from −50.3±1.7 mV to −35.3±4.8 mV (*n* = 5; *p*<0.0001, paired *t* test), and 2 out of 7 MCs died. Fig. 2 shows the activated MCs in response to 10 µM cinobufagin in an original recording from a typical MC. Similarly, bath application of 10 µM resibufogenin, which is another bufadienolide, induced irreversibly over-excitation of MCs (n = 3) and cell death (n = 2). The activation level of principal neurons evoked by cinobufagin and resibufogenin indicated that higher concentrations of bufadienolides were able to induce serious CNS toxicity in the form of excitotoxicity.

### Lower doses of cinobufagin and resibufogenin concentration-dependently increased spontaneous firing and depolarized MCs

**Figure 3:**
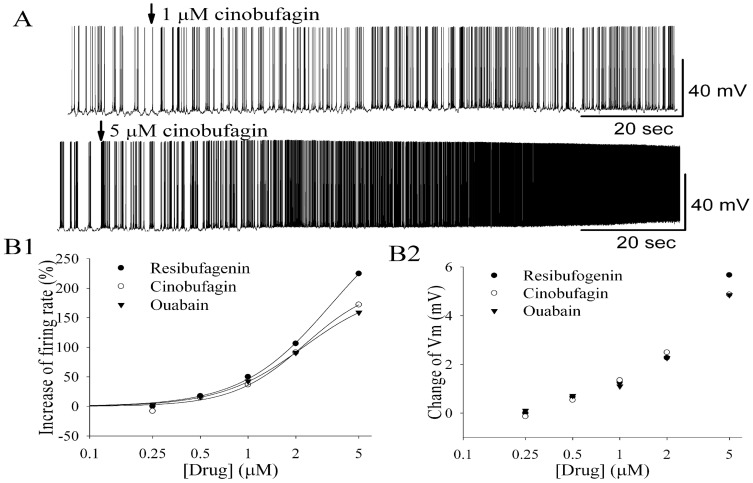
Bufadienolides and ouabain enhanced the firing activity of MCs in a concentration-dependent manner. **A** Original recording from a representative MC illustrated the increase in firing rate and depolarization following application of cinobufagin at 1 µM and 5 µM, respectively. **B1** Concentration-response curves for spiking of MCs evoked by various concentrations of bufadienolides and ouabain. The firing rates in the presence of bufadienolides and ouabain were normalized with respect to the control condition and were averaged (each point was the mean ± SEM of 3 to 7 cells). Error bars have been left out for clarity. The lines are fits for each set of data to the Hill equation: *y*  =  *Ax^n^*/(*K*_d_ *^n^* + *x^n^*), where *y* is the increase of spiking rate, *A* is the maximal increase, *K*_d_ is the apparent dissociation constant for bufadienolides, and *n* is the Hill coefficient. *K*_d_ and *A* were estimated using a Marquardt nonlinear least squares routine. **B2** Relationship between concentrations of bufadienolides or ouabain and evoked depolarization of membrane potential. Error bars were left out for clarity.

Bath application of cinobufagin at relatively lower concentrations increased the firing rate of MCs and depolarized them (Fig. 3A). Compared to control conditions, 1 µM cinobufagin reversibly increased MC firing rate from 4.4±0.6 Hz to 5.9±0.6 Hz (*n* = 5; *p*<0.001, paired *t* test). The increase in firing rate was accompanied by a depolarization of the MC membrane potential from −50.3±1.3 mV to −48.9±1.3 mV (*n* = 5; *p*<0.01, paired *t* test). No death of recorded neurons at 1 µM cinobufagin was observed. In the presence of 5 µM cinobufagin, the firing rate increased from 4.2±0.5 Hz to 10.7±1.5 Hz (*n* = 4; *p*<0.01, paired *t* test), and the membrane potential depolarized from −50.2±1.3 mV to −45.3±1.3 mV (*n* = 5; *p*<0.01, paired *t* test). One cell death out of five recorded neurons was observed at 5 µM cinobufagin.

Ouabain, a cardiac glycoside that inhibits the Na^+^/K^+^-ATPase, activated MCs, evoked an increase in firing rate of the neurons, and depolarized them with similar excitatory strength as resibufogenin and cinobufagin. Fig. 3B shows the concentration-dependent increase in firing rate of MCs and membrane potential depolarization evoked by cinobufagin, resibufogenin and ouabain. At higher concentrations, resibufogenin had a slightly stronger effect on firing rate and membrane potential of MCs compared to cinobufagin and ouabain.

### Blockade of ionotropic glutamatergic and GABAergic synaptic transmission failed to eliminate the bufadienolides-induced activation of neurons

**Figure 4:**
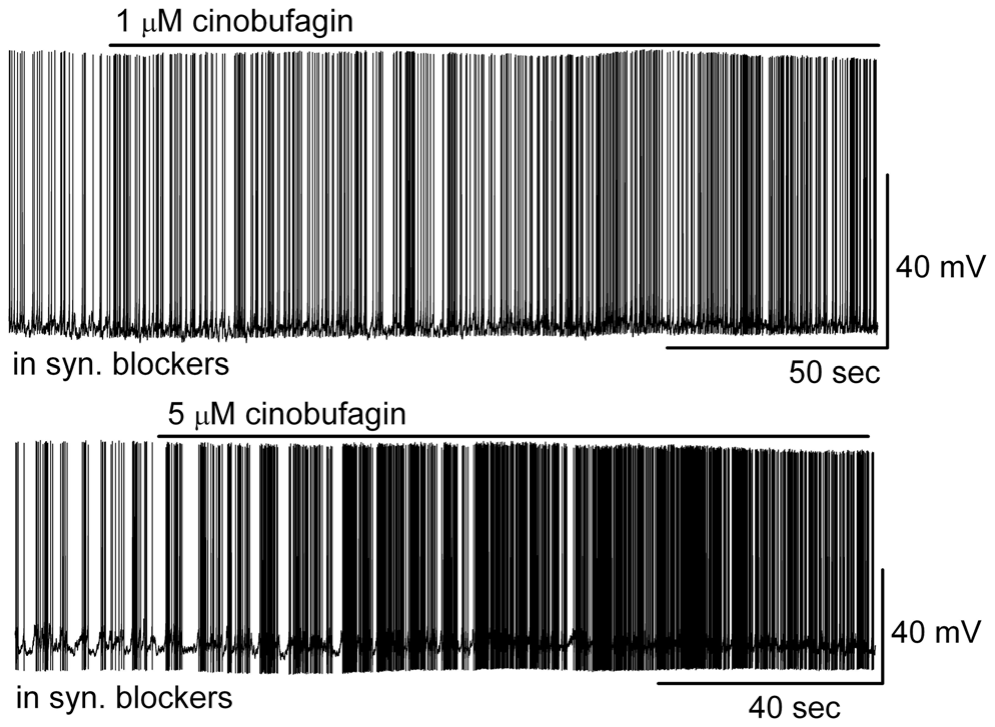
Cinobufagin activated MCs in the presence of synaptic blockers. Original recording from representative MCs illustrated the enhancement of firing rate and depolarization following application of cinobufagin. The two traces are from different MCs.

To determine whether the actions of bufadienolides on MCs were direct or indirect, we tested the effects of cinobufagin on MCs in the presence of ionotropic glutamate and GABA_A_ receptor blockers (synaptic blockers: CNQX, 10 µM; APV, 50 µM; gabazine, 5 µM). The effects of cinobufagin (1 µM, 5 µM) on firing and membrane potential of MCs persisted in the presence of synaptic blockers (Fig. 4). In synaptic blockers, 1 µM cinobufagin increased firing rate of MCs from 4.0±1.1 Hz to 5.6±1.3 Hz (*n* = 5, *p*<0.05, paired *t* test) and depolarized membrane potential from −50.8±1.4 mV to −49.2±1.4 mV (*n* = 5; *p*<0.05, paired *t* test). The effects of cinobufagin (1 µM) on MCs in synaptic blockers were not significantly different from those without blockers (*p>*0.05, determined by ANOVA and Bonferroni *post hoc* analysis). These results indicated that cinobufagin-induced activation of MCs resulted from the direct action on those cells rather than from an indirect effect on other MOB cell types mediated by synaptic transmission to MCs.

### Ouabain mimicked resibufogenin and cinobufagin-evoked excitation

It has been reported that Chan su and bufadienolides increase myocardial contractility possibly through the inhibition of ouabain-sensitive Na^+^/K^+^-ATPase [17], . To determine whether the bufadienolides effect on MCs is mediated by Na^+^/K^+^-ATPase, we tested the action of ouabain on excitability of MCs.

**Figure 5:**
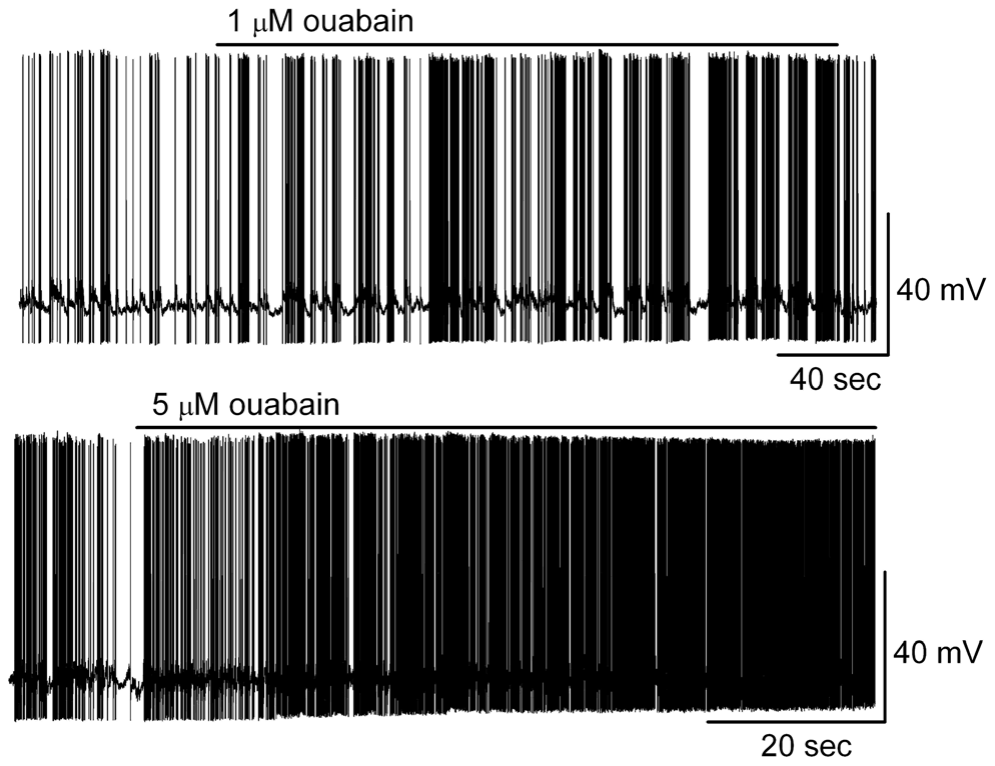
Ouabain had an excitatory effect on MCs. Original recording from representative MCs illustrated the increase in firing rate and depolarization following application of ouabain. The two traces are from different MCs.

Compared to cinobufagin and resibufogenin, we observed a similar effect of ouabain on neuronal firing rate and membrane potential (see also Fig. 3B). At equivalent levels of concentration, ouabain reversibly excited MCs in a concentration-dependent manner. At 1 µM, ouabain increased the firing rate of MCs from 4.3±1.2 Hz to 6.0±1.3 Hz (*n* = 6, *p*<0.05, paired *t* test) and depolarized the membrane potential from −49.5±1.0 mV to −48.4±1.0 mV (*n* = 5; *p*<0.05, paired *t* test). At 5 µM, ouabain showed a strong excitatory effect, similar to cinobufagin, with a firing rate increase from 4.5±1.1 Hz to 11.6±1.8 Hz (*n* = 5, *p*<0.01, paired *t* test) (Fig. 5). The similar excitatory effect on MCs suggested that bufadienolides and ouabain may share the same underlying mechanism.

### Both bufadienolides and ouabain induced inward currents

**Figure 6:**
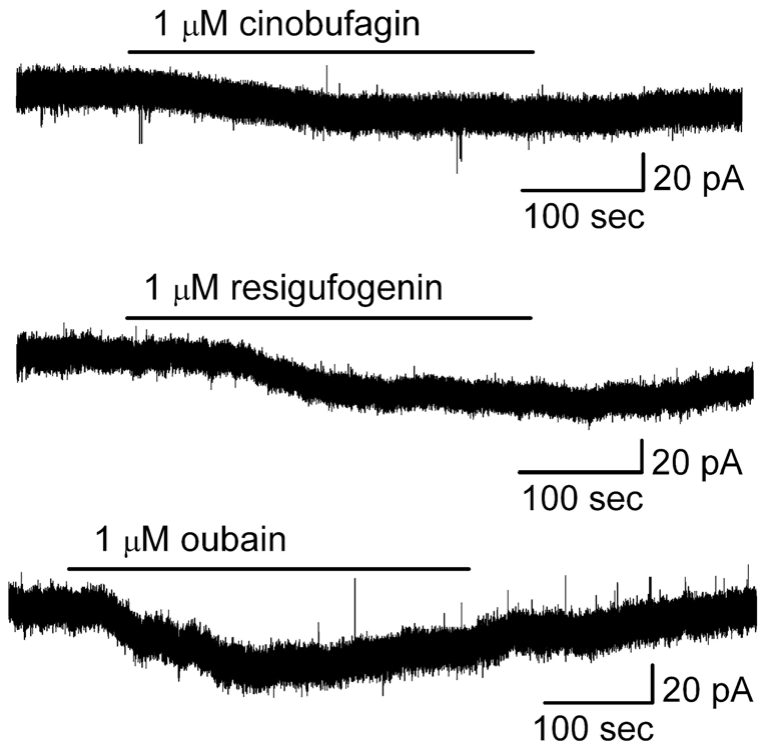
Both bufadienolides and oubain elicited inward currents in MCs. Original recordings illustrated cinobufagin-, resibufogenin- and ouabain-evoked inward currents of MCs. Data are from different cells.

The excitatory effects of bufadienolides on neurons are usually accompanied by inward currents. Evidence for an ouabain-like action of bufadienolides was obtained from measurements of MC ionic currents induced by bufadienolides and compared to those induced by ouabain. In whole-cell recording mode with low-Cl^-^ pipette solution at a holding potential of 0 mV, cinobufagin (1 µM) produced an inward current in MCs of 32.3±13.1 pA (*n* = 6; the steady state currents at 0 mV in cinobufagin were measured and subtracted from that in ACSF) (Fig. 6). Bath application of resibufogenin induced an inward currents in MCs of 33.7±8.7 pA (*n* = 5). Similarly, bath application of ouabain induced an inward currents in MCs of 29.4±7.4 pA (*n* = 5). These results suggested that bufadienolide-induced excitation in MCs may share a similar mechanism of action compared to ouabain.

### Neuronal activation by bufadienolides was independent of voltage-gated sodium channels

It was recently reported that resibufogenin can activate voltage-gated sodium (Na^+^) channels in cultured rat hippocampal neurons [32]. Possibly, activation of Na^+^ channels is another action of bufadienolides that contributes to neuronal excitation. The direct action of cinobufagin on Na^+^ channels was examined by whole-cell recording from stably transfected CHO cells that expressed the Na_v_1.2 channel isoform. The defined rat brain Na^+^ channel subtype Na_v_1.2 is known to be dominant in rat brain [37], [38].

**Figure 7:**
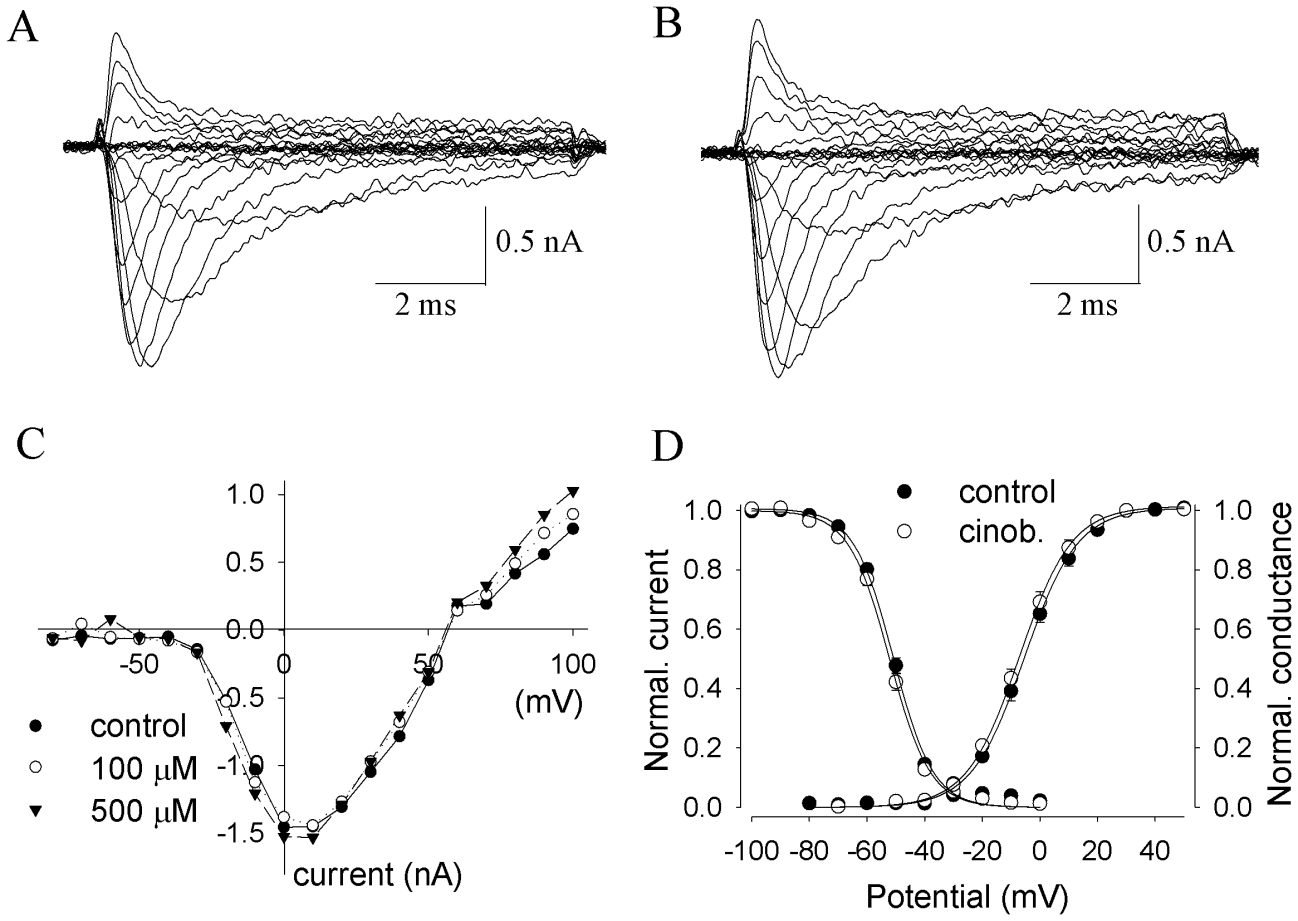
Cinobufagin had no effects on Na_v_1.2 channels. **A, B** Recording traces of activation of Na_v_1.2 currents in control (**A**) and in 100 µM cinobufagin (**B**). The currents were elicited by stepping to various depolarized potentials (ranging from −80 to +100 mV in 10-mV increments) for 9 msec, and then returning to the holding potential of −100 mV. **C** The current-voltage (I–V) relationships in control and in the presence of various concentrations of cinobufagin. Peak currents at each depolarized potential were measured. The data are from a representative cell. **D** Cinobufagin did not change Na_v_1.2 channel activation and inactivation. *Activation:* From the peak Na^+^ currents as obtained in **A** and **B**, the Na^+^ conductance values (*G*) were calculated (n = 3–5 for each point), normalized to the maximum in control and plotted as a function of membrane potentials (*V*). *Inactivation:* The voltage dependence of steady-state inactivation (*h*_∞_) was examined by applying 500-msec prepulse potentials from −100 mV to 0 mV in 10-mV increment from a holding potential of −100 mV before stepping to the test potential (0 mV) for 35 msec (n = 5 for each point). The peak current (I) for each cell was normalized with respect to the first value measured at test potential (0 mV). Conductance–voltage relationships or inactivation–voltage relationships from individual cells were fitted with a Boltzmann function, *y*  =  1/{1 + exp[(*V – V*_.5_)/*k*]} or *y*  = 1- 1/{1 + exp[(*V – V*_.5_)/*k*]}, where V is membrane potential, *V*_.5_ is the half-activation (*V*_a_) or half inactivation (*V*_h_) voltage, and *k* is a slope factor.

Bath application of cinobufagin failed to significantly influence peak currents of Na_v_1.2 or the current–voltage (I–V) relationship of Na_v_1.2 channels (Fig. 7A, B). The shape of the I–V curves was unaffected by different concentrations of cinobufagin (Fig. 7C), suggesting that cinobufagin, at the concentrations tested, had no effect on the voltage-dependent activation of Na^+^ channels, which was further analyzed in Fig. 7D. The amplitudes of the peak Na^+^ currents at various depolarized potentials were not significantly changed by different concentrations of cinobufagin (Fig. 7C). In another experiment with currents elicited by stepping to 0 mV from -100 mV holding potential, a change of current amplitude by cinobufagin was not observed either (data not shown). In experiments to determine the voltage-dependent availability of Na_v_1.2 channels (Fig. 7D), cinobufagin induced a minor left-shift of 1.3±0.3 mV (n = 5). Correspondingly, bath application with only extracellular solution also resulted in a small left-shift of 1.4±0.3 mV (n = 7). The small shifts of the steady state inactivation (*h*_∞_) of Na_v_1.2 channels in cinobufagin were not significantly different from those in control condition (*p*>0.05, determined by ANOVA and Bonferroni *post hoc* analysis), suggesting that cinobufagin did not influence the steady state inactivation (*h*_∞_).

The results obtained in cells with transfected Na_v_1.2 channels indicated that cinobufagin failed to change key parameters of neuronal Na_v_1.2 channels, suggesting that cinobufagin-induced excitation of MCs might not be mediated by activation of Na^+^ channels. The results supported the idea that bufadienolides activated CNS neurons by having an ouabain-like action.

## Discussion

We present electrophysiological evidence that bufadienolides such as cinobufagin and resibufogenin exhibited excitatory and toxic effects on neurons in the CNS. Voltage-gated Na^+^ channels were not involved in the neuronal excitatory effects. The effects of resibufogenin and cinobufagin on neuronal activity and ionic currents were mimicked by ouabain, strongly suggesting that bufadienolides activated neuron by having a ouabain-like effect, which was, most likely, mediated by inhibition of Na^+^/K^+^-ATPase rather than by activation of neuronal Na^+^ channels.

### The role of neuronal excitatory effects in pharmacological and toxic actions of bufadienolides

Chan su and bufadienolides have been reported to have both pharmacological and toxic effects in the central and peripheral nervous system *in vivo* [22]. Our results provide support for the idea that the pharmacological and toxic effects of bufadienolides on CNS neurons can result from evoked neuronal excitation and/or over-excitation.

Moderate activation of central and peripheral neurons by low concentrations of bufadienolides may be responsible for the pharmacological effects of bufadienolides such as activation of the central nervous respiratory system and their analgesic effects [20], [21]. Over-excitation of neurons in response to high concentration of bufadienolides may be responsible for the severe side-effects such as epileptic seizure, muscle cramps, shortness of breath and breathlessness, and coma that occurs by the over-doses of Liu-Shen-Wan and Chan su. High bufadienolide concentrations could also account for some pharmacological effects such as acting as a topical anesthetic [29]–[31].

Recent studies reported the action of bufadienolides on Na^+^ channels of cultured CNS neurons [32], [42]. Using whole-cell recordings from Na_v_1.2-transfected CHO cells, we did not observe any significant modification of key properties of Na_v_1.2 channels in response to cinobufagin. Even though native neurons may co-express proteins that associate with the channels and change their behavior, the failure of direct action of cinobufagin on transfected Na_v_1.2 channels suggests that cinobufagin might not have direct action on Na^+^ channels of neurons.

Our results imply that the pharmacological and toxic effects of bufadienolides or Chan su in the CNS are evoked by the excitatory actions of bufadienolides on neurons. It has been shown that intravenous injection of bufalin, cinobufagin, cinobufotalin or gamabufotalin leads to an increase in blood pressure and breathing [22]. The increase in blood pressure is thought to be related to the excitatory action on the heart. The stimulatory respiration effects and the subsequent inhibitory effects evoked by Chansu or bufadienolides in the clinic or *in vivo* suggest that the respiratory action of Chansu is CNS-related [22]. Chan su-containing herbal medicine “kyushin” also exhibits CNS-related respiratory stimulation and increase in blood pressure [29]. Our results suggest that the stimulatory respiration effect of bufadienolides may be related to the excitatory effect on central neurons which is likely mediated through inhibition of Na^+^/K^+^-ATPase.

Topical administration of medication provides a means to deliver high concentrations of medication superficially. It was shown that topical administration of Chansu exhibited strong anesthetic effects [22], [24]. High concentrations of Chan su may over-excite peripheral nerves and paralyze the neurons that transmit pain signals by disrupting Na^+^ and K^+^ gradients of the neurons. Therefore, we hypothesize that the over-excitement of the peripheral nervous system evoked by bufadienolides is mediated through inhibition of Na^+^/K^+^-ATPase and may be responsible for the topical anesthetic effects of Chan su and analgesic effect [43].

It was reported that both ouabain and digoxin presented systematic analgesic effects [15], [43]. The mechanisms underlying the systematic analgesic effect of ouabain *in vivo* may be more complicated because it required prior administration of ouabain for several consecutive days to observe the analgesic effects.

### Concentration-dependent pharmacological effects of bufadienolides suggest distinct mechanisms of action

Bufadienolides induce apoptosis mostly at concentrations of more than 10 nM in many human and other tumor cells lines [19], [44]. It has been reported that bufalin dramatically decreases the proliferation of HCCLM3 and HepG2 cell lines in a dose-dependent manner, especially when exposed to more than 10 nM bufalin [44]. Bufalin (1 µM) induces apoptosis in human tumor cells selectively *via* inhibition of the Na^+^/K^+^-ATPase [18]. It evokes a depolarization of the membrane potential in several human tumor cell lines but not in murine leukemia cells.

Over-doses of bufadienolides or Chan su-containing formulations like Kyushin, Liu-Shen-Wan have toxic effects in the heart by triggering cardiac arrhythmia [21], [29], [27], and in the CNS by triggering epileptic seizures, muscle cramps, or fatal paralysis [29]–[31]. Reports from other groups indicate that the toxic effect on the heart is related to functional modification of both cardiomyocytes and Purkinje fibers in the heart [21], [27], [29]. Our results suggest that the neurotoxic effects of bufadienolides are linked to an irreversible inhibition of neuronal activity in the CNS possibly mediated by Na^+^/K^+^-ATPase. We measured the input resistance before and after perfusion of drugs (data not shown). We found that the substances alter the input resistance of MCs. However, no statistically significant difference was found before and after the addition of drugs. The increase in input resistance by bufadienolides is thought to result from extrasynaptic “tonic” Na+/K+-ATPase inhibition. Generally, the increased input resistance will be accompanied by an increase in firing rate. Therefore, the increased input resistance may also contribute to the enhancement of neuronal excitation.

The lowest concentration of bufadienolides we tested (0.25 µM, approximately 125 ng/ml) is close to the reported concentrations of bufadienolides in human serum after ingesting Chan su. It is expected that the Chan su concentration in serum is about 40–200 ng/ml in an individual who ingests a recommended dose, while a blood level of 0.4–1.0 µg/ml is expected in a person who has ingested a moderate to large amount of Chan Su [45]. Our results suggest that concentrations above 125 ng/ml evoke dose-dependent excitation of neurons as a pharmacological effect and, thereafter, over-excitation of neurons as a toxic effect. It has been reported that a moderate concentration of Chan su (400 ng/ml) extract induces myocytes to cease beating within seconds after adding the compound [45]. It is reasonable to speculate that concentrations causing heart toxicity, at the same time have neurotoxic effects on nerve cells in the brain.

The concentration-response curve in Fig. 3B demonstrates that, at the lowest concentration of 0.25 µM, bufadienolides failed to activate MCs. Instead, the firing activity of MCs appeared to be inhibited by 0.25 µM cinobufagin, suggesting that bufadienolides may exhibit opposite effects on neuronal activity at lower concentrations of bufadienolides. This finding is consistent with reports showing that the cardiotonic steroids ouabain, digoxin, and bufadienolides all show an inverted U-shaped dose–response curve with inhibition of Na^+^/K^+^-ATPase activity at higher concentrations, while increasing Na^+^/K^+^-ATPase activity at low concentrations [9], [41]. Cardiotonic steroids like ouabain are synthesized endogenously and are active at nanomolar concentrations [9], [46]. At lower, physiological concentrations that occur *in vivo*, cardiotonic steroids could increase Na^+^/K^+^-ATPase activity [9]. Bufadienolides may similarly induce Na^+^/K^+^-ATPase activity at lower concentrations.

Chan su is often a key component found in formulations of traditional Chinese medicine such as Liu-Shen-Wan, Kyusin. Both Chinese medicines are popular traditional Chinese medicines widely used for hundreds of years as remedies for tonsillitis, sore throat, furuncle, and palpitations. The clinical use of these medicines suggests that they have an anti-inflammatory effect. The steroid ouabain is also considered an immunomodulatory molecule capable of modulating many aspects of the immune system that may participate in anti-inflammatory effects [47]. The reported serum concentrations of bufadienolides were between 0.51 nM (0.4 ng/mL) and 1.1 nM (0.88 ng/mL) in healthy volunteers after ingesting kyushin tablets or Lu-Shen-Wen pills [48], [49]. Recent studies showed that activation or modulation of Na^+^/K^+^-ATPase are involved in anti-inflammation [15], [16], [50], and in protecting hippocampal slice cultures from experimental ischemia [41]. Thus, we suggest that the activation of Na^+^/K^+^-ATPase is a possible pharmacological mechanism, at least in part, of the anti-inflammatory action of Lu-Shen-Wen. It has been recently reported that prior ouabain administration had an anti-inflammatory effect by reducing a mouse paw edema [15]. In response to inflammatory stimuli, ultralow concentrations (10^-6^ µM) of ouabain restored Na^+^/K^+^-ATPase [16]. Participation of Na^+^/K^+^-ATPase in immune responses has been reported [51]. All of these studies support the idea that nanomolar concentrations of bufadienolides have anti-inflammatory effects that contribute to the efficacy of Lu-Shen-Wen in treating tonsillitis and sore throat. Therefore, bufadienolides may exhibit distinct pharmacological effects at different levels of concentration.

## Conclusion

In conclusion, the present results suggest that bufadienolides exhibited excitatory and toxic effects on neurons in the CNS. The effects of resibufogenin and cinobufagin on neuronal activity and ionic currents were mimicked by ouabain, strongly suggesting that bufadienolides activated neuron by having an ouabain-like effect. The excitatory and toxic effects on neurons in the CNS were, most likely, mediated by inhibition of Na^+^/K^+^-ATPase rather than by activation of neuronal Na^+^ channels.
